# RECIPROCALITY OF COGNITIVE IMPAIRMENT, PAIN, AND DEPRESSION: MODELING WITHIN-PERSON EFFECTS

**DOI:** 10.1093/geroni/igae098.3348

**Published:** 2024-12-31

**Authors:** Miharu Nakanishi, Marieke Perry, Rachele Bejjani, Satoshi Yamaguchi, Satoshi Usami, Jenny van der Steen

**Affiliations:** Leiden University Medical Center, Leiden, Zuid-Holland, Netherlands; Radboud University Medical Center, Nijmegen, Gelderland, Netherlands; Impatients N.V., Amsterdam, Noord-Holland, Netherlands; Tokyo Metropolitan Institute for Geriatrics and Gerontology, Tokyo, Japan; University of Tokyo, Tokyo, Japan; Leiden University Medical Center, Leiden, Zuid-Holland, Netherlands

## Abstract

Cognitive impairment, pain, and depressive symptoms are common and interrelated factors in older adults. However, the directionality and specificity of their association remains unclarified. This study explored whether these factors prospectively increase reciprocal risk and examined the longitudinal association between these factors and quality of life (QoL). We used longitudinal data from The Older Persons and Informal Caregivers Survey Minimal Data Set (TOPICS-MDS; the Netherlands). The data of 11,582 home-dwelling older adults with or without cognitive impairment were analyzed. Older adults self-reported cognitive impairment, pain, depressive symptoms, and QoL at baseline and after 6 and 12 months of follow-up. A Random Intercept Cross-Lagged Panel Model was used to assess the prospective association between the three factors, while a multilevel linear regression analysis in a two-level random intercept model was used to examine the longitudinal associations between the three factors and QoL at the within-person level. At the within-person level, pain at 6 months was associated with subsequent depressive symptoms (β=.04, P=.024). The reverse association from depression to pain, and longitudinal associations between pain and cognitive impairment and between depressive symptoms and cognitive impairment were non-significant. Pain, depressive symptoms, and cognitive impairment showed a significant association with poor QoL 6 months later. A directional relationship was observed from pain to depressive symptoms. Pain reduction may be important in the prevention of depressive symptoms, ultimately optimizing the QoL of older adults.

